# Effectiveness of Lee Silverman Voice Treatment (LSVT-LOUD) in Parkinsonian-Type Multiple System Atrophy (MSA-P): A Case Report

**DOI:** 10.7759/cureus.73106

**Published:** 2024-11-06

**Authors:** Koji Hayashi, Rina Izumi, Namie Saito, Asuka Suzuki, Yuka Nakaya, Mamiko Sato, Yasutaka Kobayashi

**Affiliations:** 1 Department of Rehabilitation Medicine, Fukui General Hospital, Fukui, JPN; 2 Graduate School of Health Science, Fukui Health Science University, Fukui, JPN

**Keywords:** dysarthria, dysphagia, lee silverman voice treatment, multiple system atrophy (msa), parkinsonism

## Abstract

We describe a case of Parkinsonian-type multiple system atrophy (MSA-P) treated with Lee Silverman Voice Treatment (LSVT-LOUD; LSVT Global, Inc., Phoenix, AZ, USA). At age 73, the patient developed motor symptoms, including gait disturbances with a tendency to fall, as well as swallowing difficulties and impaired dexterity in his right hand, prompting a visit to our hospital. Brain magnetic resonance imaging (MRI) revealed atrophy in the cerebellum and brainstem, particularly in the pons, along with enlargement of the fourth ventricle; however, the "cross sign" on the pons was not clearly visible. Dopamine transporter single-photon emission computed tomography (DAT-SPECT) showed decreased nuclide accumulation in the striatum. Additionally, ^123^I-MIBG cardiac scintigraphy demonstrated preserved nuclide accumulation in the heart. L-dopa challenge tests were conducted, but no significant improvement in motor symptoms was observed. Based on these findings, he was diagnosed with MSA-P. Over the following years, his condition progressively worsened, with increasing orthostatic hypotension, dysphagia, and falls. Various treatments, including anti-Parkinson’s medications and vasopressors, provided little relief. At age 75, due to severe dysphagia and hoarseness, he was admitted for LSVT-LOUD therapy. After LSVT-LOUD treatment, the patient improved voice volume, tongue pressures, alternating motion rates of "pa," "ta," and "ka," and the Frontal Assessment Battery (FAB) score. These results suggest that LSVT-LOUD may positively impact both speech and swallowing functions, as well as frontal lobe function. Larger studies are needed to validate these results.

## Introduction

Multiple system atrophy (MSA) is a rare, progressive neurodegenerative disorder that typically begins in adulthood (after the age of 30) and is characterized by a combination of Parkinsonism, cerebellar dysfunction, and autonomic failure [1]. Historically, three conditions were documented separately: olivopontocerebellar atrophy, striatonigral degeneration, and Shy-Drager syndrome (SDS) [1,2]. However, in 1969, Graham and Oppenheimer coined the term "MSA" to encompass all three conditions [2]. Today, olivopontocerebellar atrophy is referred to as cerebellar-type multiple system atrophy (MSA-C), while striatonigral degeneration is known as Parkinsonian-type multiple system atrophy (MSA-P). Although symptoms progress, there is no cure for MSA, and the average life expectancy is reported to be seven to nine years [1,3].

Patients with MSA often experience laryngeal dysfunction, leading to laryngeal stridor [4]. This stridor results from the inability to adduct the vocal cords, primarily due to atrophy of the posterior cricoarytenoid muscles in the larynx [5]. Additionally, individuals with MSA exhibit pharyngeal dysfunction, including difficulties with tongue base contact against the pharyngeal wall, initiating swallowing, pharyngeal pooling, laryngeal closure and elevation, as well as penetration, aspiration, and food residue in the valleculae and/or pyriform sinus after swallowing [6].

Developed in the 1980s, Lee Silverman Voice Treatment (LSVT-LOUD; LSVT Global, Inc., Phoenix, AZ, USA) has become widely recognized as the most thoroughly researched and effective communication therapy for individuals with Parkinson’s disease (PD) [7]. LSVT-LOUD aims to enhance vocal fold adduction and increase vocal loudness through high-effort, intensive training [8]. Several randomized controlled trials in the literature have demonstrated that LSVT-LOUD improves vocal intelligibility, addresses speech problems, and enhances quality of life [8]. At present, LSVT research methods are standardized, and the reliability of outcome measures has improved [8]. Among conditions that cause Parkinsonism other than PD, there are two studies on the use of LSVT-LOUD in progressive supranuclear palsy (PSP) [9,10], and only one study on its use in MSA-C [11]. However, no reports exist on the use of LSVT-LOUD for MSA-P. In this report, we present a case of MSA-P treated with LSVT-LOUD and discuss its efficacy, highlighting the parameters that improved or remained stable.

## Case presentation

**Figure 1:**
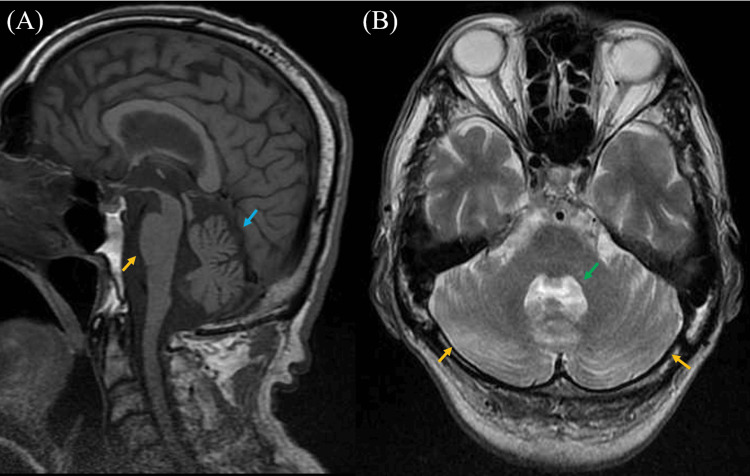
Results of brain MRI (A) T1-weighted brain MRI showing atrophy of the basilar pons (yellow arrow) and cerebellum (blue arrow). (B) T2-weighted brain MRI showing atrophy of the cerebellum (yellow arrows) and enlargement of the fourth ventricle (green arrow). MRI: Magnetic resonance imaging

**Figure 2:**
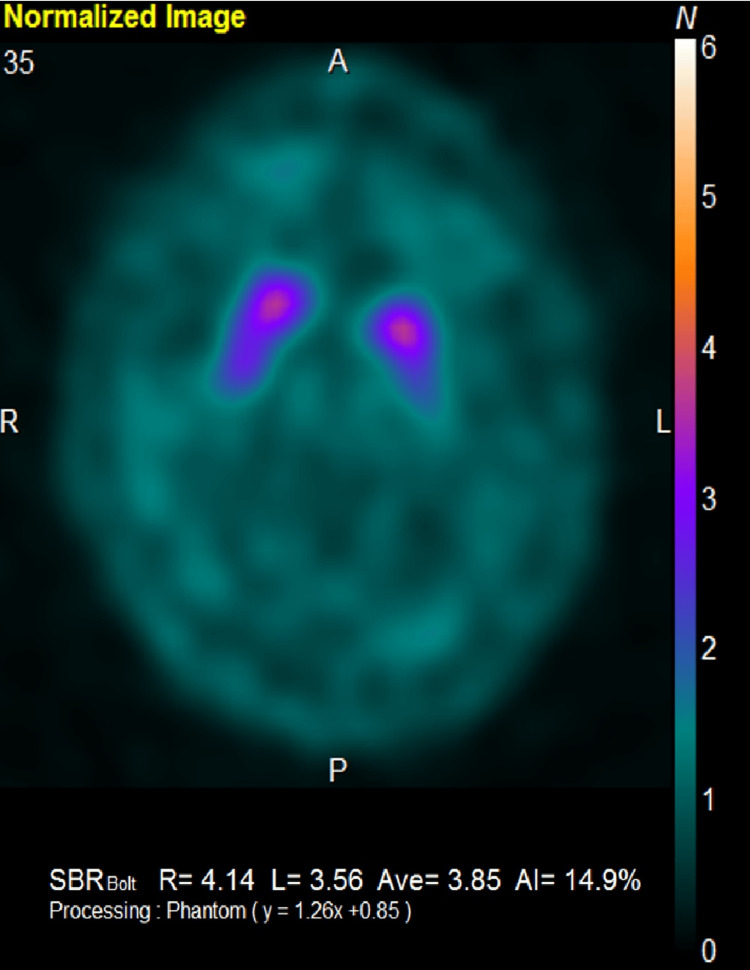
Results of the DAT-SPECT scan The DAT-SPECT scan revealed an asymmetric reduction in radiotracer uptake within the striatum. The specific binding ratio was 4.14 on the right and 3.16 on the left, respectively. DAT-SPECT: Dopamine transporter single-photon emission computed tomography

**Figure 3:**
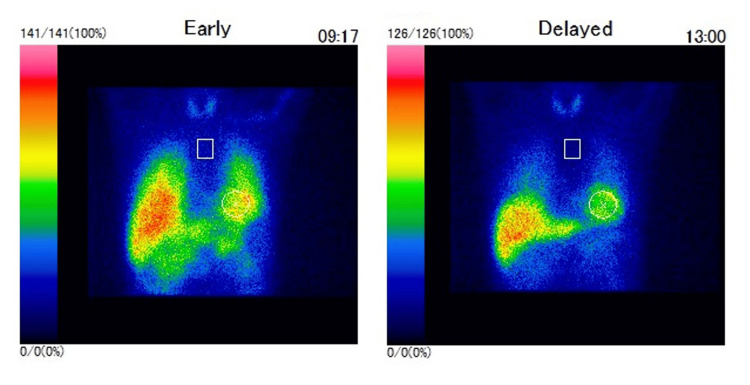
Results of the 123I-MIBG cardiac scintigraphy The^ 123^I-MIBG cardiac scintigraphy demonstrated a preserved accumulation of nuclides in the heart. The heart-to-mediastinum ratio (H/M ratio) mean values were 3.68 for the early (15-minute) acquisition and 4.55 for the late (240-minute) acquisition.

A 70-year-old Japanese man presented with hoarseness and was seen at the Otorhinolaryngology Department of another hospital, where he was diagnosed with unilateral vocal cord paralysis, although the specifics of the diagnosis were unclear. Around the same time, he reported lumbar spondylosis and neuropathic pain in both extremities and visited the Orthopedics Department at our hospital. He was prescribed several pain medications, including amitriptyline, tramadol, diazepam, and tizanidine, but these provided limited relief. At age 73, he developed gait disturbances with a tendency to fall, along with swallowing difficulties and impaired dexterity in his right hand, and subsequently visited our department. Neurological examinations revealed positive findings for applause and Myerson's sign, saccadic eye movements, dysarthria, a small voice, hoarseness, subjective difficulty swallowing, grade 4 muscle strength in the right iliopsoas muscle, hyperreflexia in the right limbs, small amplitude in bilateral finger taps, decomposition of movement on the finger-nose-finger test, a wide-based gait, brachybasia, constipation, difficulty urinating and frequent urination, and lightheadedness upon standing. Muscle rigidity was unremarkable, and both the thumb search test and Romberg's sign were negative. Brain magnetic resonance imaging (MRI) revealed atrophy in the cerebellum and brainstem, particularly in the pons, along with enlargement of the fourth ventricle (Figure 1). The "cross sign" on the pons was not clearly visible. The dopamine transporter single-photon emission computed tomography (DAT-SPECT) scan revealed an asymmetric reduction in radiotracer uptake within the striatum (Figure 2). Furthermore, ^123^I-MIBG cardiac scintigraphy demonstrated a preserved accumulation of nuclides in the heart (Figure 3). L-dopa challenge tests were performed, but there was no significant improvement in motor symptoms. Based on these findings, he was diagnosed with MSA-P.

Despite regular outpatient visits, he experienced a gradual increase in falls and worsening orthostatic hypotension. Although vasopressors were prescribed, he developed supine hypertension. Nausea and epigastric discomfort worsened, leading to a reduction in food intake. Six months after his initial visit, muscle rigidity, predominantly on the right side, was noted during routine outpatient evaluations. Anti-Parkinson's medications, including ropinirole, trihexyphenidyl, and droxidopa, were introduced experimentally but proved ineffective. One year after his initial visit, at age 74, he was admitted to our hospital due to immobility and worsening Parkinsonian symptoms following frequent falls. He was hospitalized for one month and then discharged home, but his orthostatic hypotension and gait disturbances worsened. Additionally, dysphagia and hoarseness became more pronounced. He developed leg edema, which was treated with furosemide, and his anorexia improved with the administration of mirtazapine. Due to severe MSA-related dysphagia and reduced vocal volume, he was admitted to the hospital for LSVT-LOUD therapy at age 75.

On admission, the neurological examination revealed a masked face, a positive Myerson’s sign, saccadic eye movements, breathy hoarseness, a very soft voice, rough speech, drooling from the corners of the mouth, a forward-leaning posture, occasional finger tremors, right-sided cogwheel rigidity, limbs and truncal ataxia, and loss of coordination on the hand pronation test, finger-nose-finger test, and heel-knee test. The patient also demonstrated retropulsion, brachybasia, a frozen gait, severe orthostatic hypotension with a ≥20/10 mmHg blood pressure drop immediately after standing up, and constipation. Muscle strength was relatively preserved, with grades 4 to 5 in all four extremities. The patient was assessed as stage IV on the Hoehn and Yahr Scale. LSVT-LOUD, a speech therapy designed to improve vocal cord adduction and increase voice intensity, was administered by a certified speech-language pathologist. The patient underwent 16 sessions over the course of one month, with sessions held four days per week. Additionally, the patient was instructed to practice independently for 5-10 minutes daily and for 10-15 minutes on days without therapy. Voice volume was measured using the DIGITAL SOUND LEVEL METER (GS-04; be-s Co. Ltd., Osaka, Japan) in a quiet room with an ambient noise of less than 50 dB. Tongue pressure was measured using the JMS tongue pressure measurement device (TPM-01; JMS Co. Ltd., Hiroshima, Japan).

The pre- and post-treatment evaluations of LSVT-LOUD are shown in Table 1. Voice volume, tongue pressures, and alternating motion rates of "pa," "ta," and "ka" improved drastically, enabling medical professionals to hear the patient's voice more clearly after treatment. Additionally, the Frontal Assessment Battery (FAB) score improved from 10 to 14. However, there was no effect on cognitive function, as measured by the Revised Hasegawa Dementia Scale (HDS-R) and the Mini-Mental State Examination (MMSE), nor on the Repetitive Saliva Swallowing Test (RSST). The patient was discharged from our hospital on day 30 after admission.

**Table 1:** Changes in parameters between pre- and post-LSVT-LOUD therapy. LSVT-LOUD: Lee Silverman Voice Treatment

Parameters	Pre-LSVT-LOUD	Post-LSVT-LOUD
The Revised Hasegawa's Dementia Scale (total score, maximum 30)	27	26
The Mini-Mental State Examination (total score, maximum 30)	27	29
The Frontal Assessment Battery (total score, maximum 18)	10	14
Alternating motion rates "pa" per second	1.8	5.8
Alternating motion rates "ta" per second	2.0	6.0
Alternating motion rates "ka" per second	2.6	3.6
Sound pressure of the high tone vowel (dB)	70.0	79.9
Sound pressure of the low tone vowel (dB)	70.0	80.3
Sound pressure of the moderate tone vowel (dB)	68.0	77.8
Repetitive saliva swallowing test	1.0	1.0

## Discussion

We present a case of MSA-P treated with LSVT-LOUD. The patient exhibited severe autonomic symptoms, including orthostatic hypotension, poor responsiveness to L-dopa, and cerebellar syndrome affecting both the limbs and trunk, resulting in ataxia. Motor symptoms began at age 73, with rapid progression over two years, leading to severe postural instability, dysphagia, and speech impairment. Brain MRI revealed atrophy of both the pons and cerebellum. The DAT-SPECT and ^123^I-MIBG cardiac scintigraphy play crucial roles in the differential diagnosis of Parkinsonism, particularly in distinguishing between MSA and PD [12,13]. DAT-SPECT evaluates the presynaptic dopaminergic function and typically shows reduced uptake in both MSA and PD, making it useful for confirming dopaminergic degeneration [12]. However, meta-iodobenzylguanidine (MIBG) myocardial scintigraphy is especially valuable because it reveals cardiac sympathetic denervation, which is markedly reduced in PD but usually preserved or only mildly affected in MSA [13]. Our patient's results from these nuclear tests were consistent with MSA.

In 2022, the Movement Disorders Society introduced updated diagnostic criteria for MSA, outlining four levels of certainty: neuropathologically confirmed (postmortem), clinically established, clinically probable, and possible prodromal MSA [14]. Based on these criteria, our patient was diagnosed with clinically established MSA. In our case, Parkinsonism initially appeared, followed by the development of cerebellar symptoms. Additionally, the "hot cross bun sign" on MRI, commonly seen in the pons, is useful for distinguishing between clinical subtypes of MSA, as it typically appears about five years earlier in MSA-C than in MSA-P [15]. This sign was not observed in our patient. Based on clinical and radiological findings, this case was diagnosed as MSA-P. As a result of LSVT-LOUD treatment, the patient improved voice volume, tongue pressures, alternating motion rates of "pa," "ta," and "ka," and the FAB score. He and his family were pleased with the increased volume and clarity of his voice, which made communication much easier.

Previous studies assessing the effectiveness of LSVT-LOUD in MSA-C have shown significant improvements in various speech and swallowing measures, including maximum phonation time (MPT), voice intensity, and the pharyngeal phase on the videofluoroscopic dysphagia scale [11]. These improvements have been observed not only in MSA-C but also in conditions such as PD and PSP [10,16]. The improvements are believed to result from LSVT-LOUD enhancing neural pathways, which, in turn, boosts the strength and activation of the laryngopharyngeal muscles [17]. Specifically, the suprahyoid muscles, responsible for elevating and protecting the larynx, are likely engaged by the high-intensity, repetitive exercises involving volume, pitch, and speech in LSVT-LOUD [16]. This activation may contribute to an overall increase in daily swallowing function [16]. The alternating motion rates of "pa," "ta," and "ka" are measured through oral diadochokinesis (ODK) [18], and recent studies have suggested that reduced ODK is associated with impaired swallowing function [18]. Although our case did not directly assess swallowing function, it appears to align with findings from previous reports.

In our study, we observed an improvement in the FAB score, which is associated with frontal lobe functions, particularly executive function [19]. Additionally, previous research has shown that MSA significantly reduces FAB scores compared to PD [20]. To our knowledge, no other studies of LSVT-LOUD have examined FAB scores. While the precise mechanisms behind this improvement remain unclear, a ^15^O-H_2_O PET study conducted before and after LSVT-LOUD treatment showed increased activity in the frontal lobes, including the anterior insular cortex and dorsolateral prefrontal cortex [21]. Since the dorsolateral prefrontal cortex is involved in executive function [22], we hypothesize that LSVT-LOUD may have a positive effect on frontal lobe function, particularly in executive function.

A limitation of this study is that it involves only a single case, which means the results cannot be generalized to other cases of MSA-P. Larger studies are needed to replicate and validate these findings.

## Conclusions

To the best of our knowledge, this is the first report of MSA-P treated with LSVT-LOUD. The patient showed improvements in voice volume, tongue pressure, and alternating motion rates for "pa," "ta," and "ka," as well as in the FAB score following LSVT-LOUD. These results suggest that LSVT-LOUD may positively impact both speech and swallowing functions, as well as frontal lobe function. However, the current data pool on LSVT-LOUD's application in MSA-P remains limited, highlighting the need for larger, controlled studies to confirm its efficacy. Future research should include randomized controlled trials with larger sample sizes and long-term follow-ups to better understand the extent of its benefits and the mechanisms underlying these improvements.

## References

[1] Goh YY, Saunders E, Pavey S, Rushton E, Quinn N, Houlden H, Chelban V (2023). Multiple system atrophy. Pract Neurol.

[2] Graham JG, Oppenheimer DR (1969). Orthostatic hypotension and nicotine sensitivity in a case of multiple system atrophy. J Neurol Neurosurg Psychiatry.

[3] Poewe W, Stankovic I, Halliday G (2022). Multiple system atrophy. Nat Rev Dis Primers.

[4] Bannister R, Gibson W, Michaels L, Oppenheimer DR (1981). Laryngeal abductor paralysis in multiple system atrophy. A report on three necropsied cases, with observations on the laryngeal muscles and the nuclei ambigui. Brain.

[5] Guindi GM, Michaels L, Bannister R, Gibson W (1981). Pathology of the intrinsic muscles of the larynx. Clin Otolaryngol Allied Sci.

[6] Smith C, Bryan K (1992). Speech and swallowing dysfunction in multisystem atrophy. Clin Rehabil.

[7] Bryans LA, Palmer AD, Anderson S, Schindler J, Graville DJ (2021). The impact of Lee Silverman Voice Treatment (LSVT LOUD®) on voice, communication, and participation: findings from a prospective, longitudinal study. J Commun Disord.

[8] Papadopoulos A, Voniati L, Ziavra N, Tafiadis D (2024). The effectiveness of Lee Silverman Voice Treatment (LSVT LOUD) on children's speech and voice: a scoping review. Brain Sci.

[9] Sale P, Castiglioni D, De Pandis MF (2015). The Lee Silverman Voice Treatment (LSVT®) speech therapy in progressive supranuclear palsy. Eur J Phys Rehabil Med.

[10] Nozaki S, Fujiu-Kurachi M, Tanimura T (2021). Effects of Lee Silverman Voice Treatment (LSVT LOUD) on swallowing in patients with progressive supranuclear palsy: a pilot study. Prog Rehabil Med.

[11] Park A, Jang SJ, Kim NE, Kim TH, Sohn YH, Kim H, Cho SR (2022). Swallowing outcomes following voice therapy in multiple system atrophy with dysphagia: comparison of treatment efficacy with Parkinson’s disease. Dysphagia.

[12] Pagano G, Niccolini F, Politis M (2016). Imaging in Parkinson's disease. Clin Med (Lond).

[13] Courbon F, Brefel-Courbon C, Thalamas C (2003). Cardiac MIBG scintigraphy is a sensitive tool for detecting cardiac sympathetic denervation in Parkinson's disease. Mov Disord.

[14] Wenning GK, Stankovic I, Vignatelli L (2022). The movement disorder society criteria for the diagnosis of multiple system atrophy. Mov Disord.

[15] Horimoto Y, Aiba I, Yasuda T (2002). Longitudinal MRI study of multiple system atrophy - when do the findings appear, and what is the course?. J Neurol.

[16] El Sharkawi A, Ramig L, Logemann JA (2002). Swallowing and voice effects of Lee Silverman Voice Treatment (LSVT): a pilot study. J Neurol Neurosurg Psychiatry.

[17] Fox CM, Ramig LO, Ciucci MR, Sapir S, McFarland DH, Farley BG (2006). The science and practice of LSVT/LOUD: neural plasticity-principled approach to treating individuals with Parkinson disease and other neurological disorders. Semin Speech Lang.

[18] Min SY, Pang NS, Kim YR, Jeong SA, Jung BY (2024). Factors associated with age-related changes in oral diadochokinesis and masticatory function in healthy old adults. BMC Oral Health.

[19] Moreira HS, Costa AS, Castro SL, Lima CF, Vicente SG (2017). Assessing executive dysfunction in neurodegenerative disorders: a critical review of brief neuropsychological tools. Front Aging Neurosci.

[20] Paviour DC, Winterburn D, Simmonds S (2005). Can the frontal assessment battery (FAB) differentiate bradykinetic rigid syndromes? Relation of the FAB to formal neuropsychological testing. Neurocase.

[21] Liotti M, Ramig LO, Vogel D (2003). Hypophonia in Parkinson's disease: neural correlates of voice treatment revealed by PET. Neurology.

[22] Shaked D, Katzel LI, Seliger SL (2018). Dorsolateral prefrontal cortex volume as a mediator between socioeconomic status and executive function. Neuropsychology.

